# Inhibitory effect of (−)-epigallocatechin-3-gallate and bleomycin on human pancreatic cancer MiaPaca-2 cell growth

**DOI:** 10.1186/s13027-015-0016-y

**Published:** 2015-07-29

**Authors:** Sabrina Bimonte, Maddalena Leongito, Antonio Barbieri, Vitale Del Vecchio, Massimiliano Barbieri, Vittorio Albino, Mauro Piccirillo, Alfonso Amore, Raimondo Di Giacomo, Aurelio Nasto, Vincenza Granata, Antonella Petrillo, Claudio Arra, Francesco Izzo

**Affiliations:** Division of Abdominal Surgical Oncology, Hepatobiliary Unit, Istituto Nazionale per lo studio e la cura dei Tumori “Fondazione G. Pascale”, − IRCCS - Via Mariano Semmola, 80131 Naples, Italy; S.S.D Sperimentazione Animale, Istituto Nazionale per lo studio e la cura dei Tumori “Fondazione G. Pascale”, − IRCCS - Via Mariano Semmola, 80131 Naples, Italy; Division of Radiology, Istituto Nazionale per lo studio e la cura dei Tumori “Fondazione G. Pascale”, − IRCCS - Via Mariano Semmola, 80131 Naples, Italy

**Keywords:** (−)-Epigallocatechin-3-gallate, Pancreatic cancer, Bleomycin, Cell proliferation, Apoptosis

## Abstract

**Background:**

Human pancreatic cancer is currently one of the deadliest cancers with high mortality rate. It has been previously shown that (−)-epigallocatechin-3-gallate (EGCG), the most abundant catechin found in green tea, has showed suppressive effects on human pancreatic cancer cells. Bleomycin, (BLM), an anti-cancer chemotherapeutic drug that induces DNA damage, has antitumor effects by induction of apoptosis in several cancer cell lines and also in pancreatic cancer cells. The present study investigated for the first time, the inhibitory effect of EGCG and BLM on pancreatic cancer cell growth.

**Methods:**

Using the pancreatic cancer cell lines MIA PaCa-2 cells the efficacy and synergism of EGCG and BLM were evaluated by *in vitro* tests. Inhibition of cell proliferation was determined by MTT assay. Mitochondrial depolarization was performed with JC-1 probe. Viability and apoptosis were determined by Flow Cytometry with annexin V, propidium iodide staining and DNA fragmentation assay.

**Results:**

Cell proliferation assay revealed significant additive inhibitory effects with combination of EGCG and BLM at 72 h in a dose dependent manner. The combination of EGCG and BLM induced cell cycle S-phase arrest and mitochondrial depolarization. Viability, apoptosis and DNA fragmentation assay indicated that the combination of EGCG and bleomycin potentiated apoptosis.

**Conclusions:**

Our results indicate that EGCG and BLM have additive anti-proliferative effects in *vitro* by induction of apoptosis of MIA PaCa-2 cells. This combination could represent a new strategy with potential advantages for treatment of pancreatic cancer. To date, this is the first report published of the inhibitory effect of EGCG and BLM on human pancreatic cancer MIA Paca-2 cell growth.

## Background

Human pancreatic cancer is currently one of the deadliest cancers with high mortality rate [1]. Only a small fraction of patients at presentation have resectable disease because of either presence of distant metastases or locally advanced disease involving neighboring vasculature and associated perineural invasion [2]. Radical resection is still the only curative treatment for pancreatic cancer, but it is generally accepted that a multimodality strategy is necessary for its management. Conventional treatment of pancreatic cancer is a combination of surgical resection, chemotherapy, and radiotherapy [3–5] although chemotherapeutic agents have not been very effective for human pancreatic adenocarcinoma. The poor prognosis of pancreatic cancer is due to its tendency for late presentation, aggressive local invasion, early metastases, and poor response to chemotherapy. Currently, the best chemotherapeutic agent available for treatment of pancreatic cancer is gemcitabine [6]. However, gemcitabine treatment is associated with several side effects and drug resistance [5]. Thus novel strategies involving less toxic agents for treatment of pancreatic cancer have been developed [7–10]. Integrative oncology is a new focus in cancer research that involves association of natural compounds, as nontoxic tools, with traditional chemotherapy [11]. The most accepted compounds for chemoprevention in humans are substances present in diet. It has been showed that green tea catechins, inhibits tumor growth at different organ site [12–14]. (−)-Epigallocatechin-3-gallate (EGCG) is the most abundant catechin found in green tea and it is used in cancer prevention and treatment. It has been demonstrated that this compound, is able to inhibit cell growth and to induce apoptosis in cancer cells without normal cellular damage [15, 16]. EGCG also inhibits angiogenesis, possibly through the inhibition of proangiogenic factors, including vascular endothelial growth factor (VEGF) [17]. Even if the chemopreventive activity of EGCG was largely studied, the underlying mechanisms are not yet completely clear. Recently, it has been showed that EGCG inhibits pancreatic cancer orthotopic tumor growth, angiogenesis and metastasis associated with inhibition of ERK and PI3K/AKT pathways and activation of FKHRL1/FOXO3a [18]. In addition the antiproliferative effects of EGCG on pancreatic cancer cell growth *in vitro*, are potentiated by treatment with pterostilbene, a stilbenoid derived from blueberries [11]. Bleomycin (BLM) is a glycopeptid drug originally isolated from *Streptomyces verticillus* [19, 20] is clinically used for cancer therapy [20, 21]. The bleomycins are a family of glycopeptide-derived antitumor antibiotics used clinically for the treatment of squamous cell carcinomas and malignant lymphomas [22–24]. Their antitumor activity is due to selective oxidative cleavage of 5′-GC-3′ and 5′-GT-3′ sequences in DNA, and possibly also to RNA oxidative degradation [25–31]. BLM plays several roles in a network involving multiple pathways for chromosome remodeling, DNA/RNA binding and processing, signal transduction, DNA repair, cell cycle, and apoptosis [30, 32, 33]. It has been demonstrated that BLM causes DNA damage and kill HepG2 cells, by apoptosis. The same effect, it has been observed in PANC-1 and in HPAC pancreatic cancer cells [34]. Recently, it has been demonstrated that the anticancer effects of BLM on MIA PaCa-2 cells is potentiated by electrochemotherapy i*n vitro* [35]. The present study investigated for the first time, the inhibitory effect of EGCG and BLM on MIA PaCa-2 pancreatic cancer cell growth. Our results indicate that EGCG and BLM have additive anti-proliferative effects in *vitro* by induction of apoptosis of MIA PaCa-2 cells. This combination could represent a new strategy with potential advantages for treatment of pancreatic cancer.

## Results

### Effects of EGCG and BLM on MIA PaCa-2 cell proliferation

**Fig. 1:**
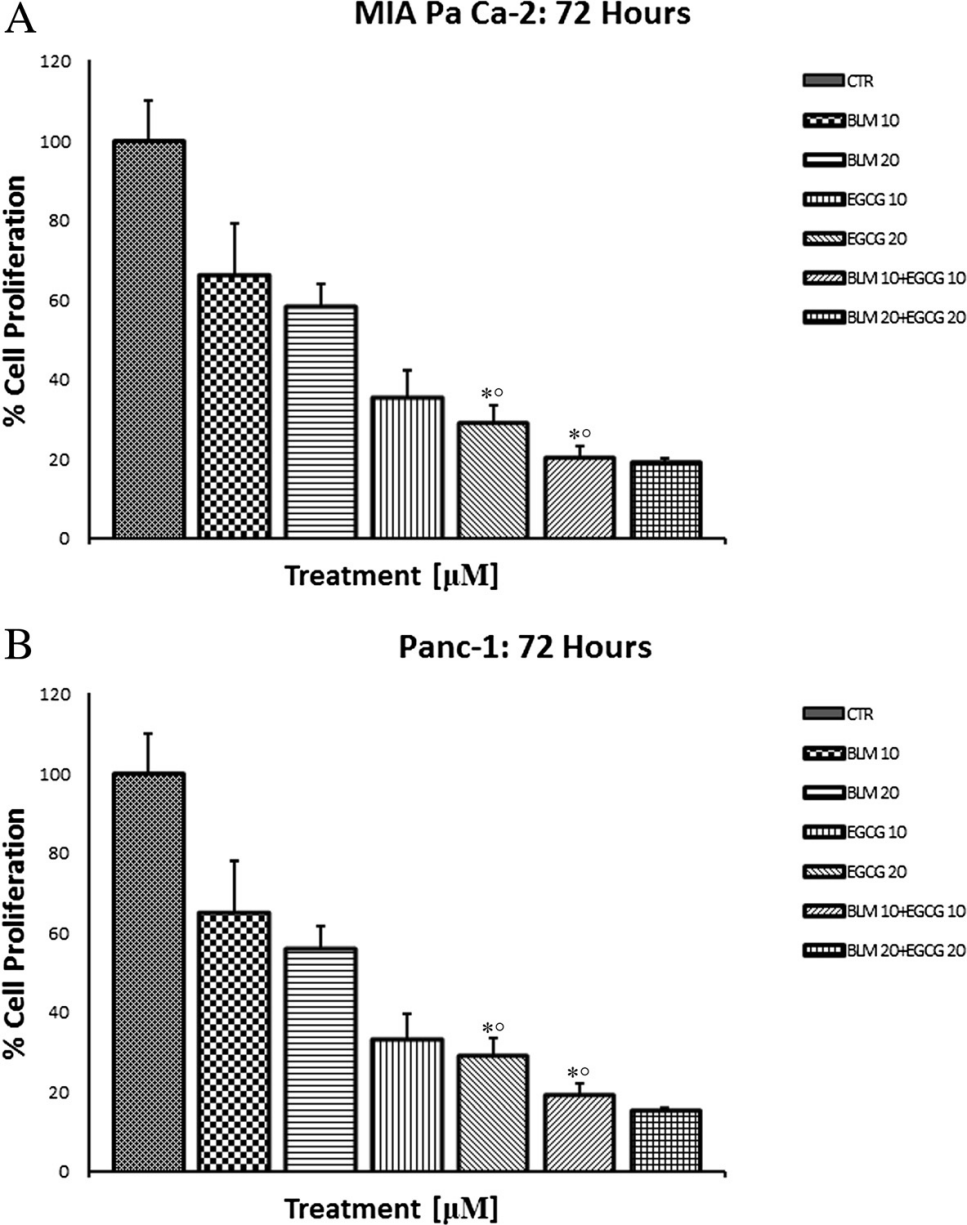
Effects of EGCG and BLM on MIA PaCa-2 cell proliferation. MTT cell viability assays showing reduction in cellular proliferation from treatment with EGCG alone, BLM alone, and both agents in MIA PaCa-2 cell lines **a** and Panc-1 cell lines **b** in *vitro* at 48 h of incubation. Data presented as mean ± standard deviation. ^∗^P < 0.001, by analysis of variance, compared with control

**Fig. 2:**
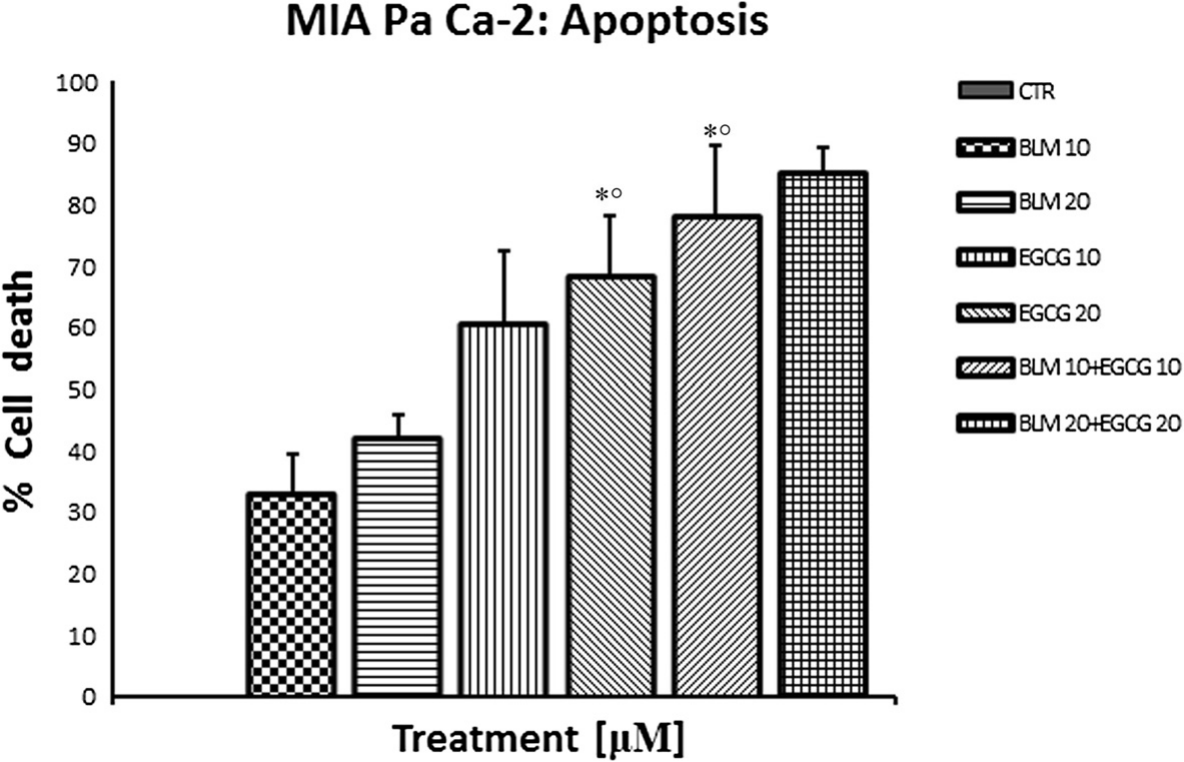
Effects of EGCG and BLM on MIA PaCa-2 apoptosis. *In vitro* apoptosis assay by flow cytometry indicated that EGCG and BLM (20 *μ*M) enhanced apoptosis in MIA PaCa-2 cells (*P value < 0.0001)

We first determined whether EGCG and BLM inhibited the proliferation of human pancreatic cancer cells by performing MTT assays on MIA PaCa-2 cells. MTT assay demonstrated significant reductions in cellular proliferation at all treatments levels after 72 h of incubation (P = 0.001) (Fig. 1a). This result was also confirmed on Panc-1 cells (Fig. 1b). We also performed *in vitro* apoptosis assay by flow cytometry, to assess if the combination treatments enhanced the apoptosis in pancreatic cancer cells. Our results showed that the percentage of apoptosis of MIA PaCa-2 cells treated with EGCG (20 μM) and BLM (20 μM) was higher with respect to controls and to single treatments (Fig. 2). Taken together, our results suggest that the combination of EGCG and BLM inhibits proliferation and enhanced apoptosis of MIA PaCa-2 cells.

### Effects of EGCG and BLM on MIA PaCa-2 on cell cycle and mitochondrial depolarization

We also performed cell cycle analysis of MIA PaCa-2 cells treated with EGCG alone, BLM alone, and both agents together. Results summarized in Table 1, showed that normal cell cycle progression was disrupted after 24 h and 48 h of incubation. In particular, treatment with BLM (20 μM) combined with EGCG (20 μM) caused an increased percentage of MIA PaCa-2 cells to arrest in the S-phase compared to controls. We also tested the mitochondrial depolarization in MIA PaCa-2 cells. Mitochondrial depolarization of JC-1-labeled MIA PaCa-2 cell was observed after 30 min-treatments. Results showed that the combination treatment (20 μM EGCG plus 20 μM BLM) leads to an increase in the percentage of depolarized cells (15,2 %) compared to controls (5,00 %). Table 1 and 2

**Table 1:** Cell cycle analysis with EGCG and BLM in MIA PaCa-2 cells

Treatment	G_0_/G_1_	S	G_2_/M
MIA PaCa-2			
Control	63	12	25
DMSO	64	12	25
20 μM BLM	45	26	27
20 μM EGCG	67	10	22
20 μM EGCG plus 20 μM BLM	50	20	21

Cell cycle analysis MIA PaCa-2 cell lines when treated with EGCG alone, BLM alone, and both agents together

**Table 2:** Mitochondrial depolarization with EGCG and bleomycin in MIA PaCa-2 cells

Treatment	Normal (%)	Depolarized(%)
MIA PaCa-2		
Positive control	80.8	27.4
DMSO	93.0	4.93
20 μM BLM	91.0	11.3
20 μM EGCG	87.8	13.6
20 μM EGCG plus BLM	80.9	26.2

EGCG = (-)-epigallocatechin-3-gallate; DMSO = dimethyl sulfoxide

Data presented as percentage of normal and depolarized cells

Mitochondrial depolarization of JC-1–labeled MIA PaCa-2 cells after 30-min treatments

### Effects of EGCG and BLM on MIA PaCa-2 cells programmed cell death

**Fig. 3:**
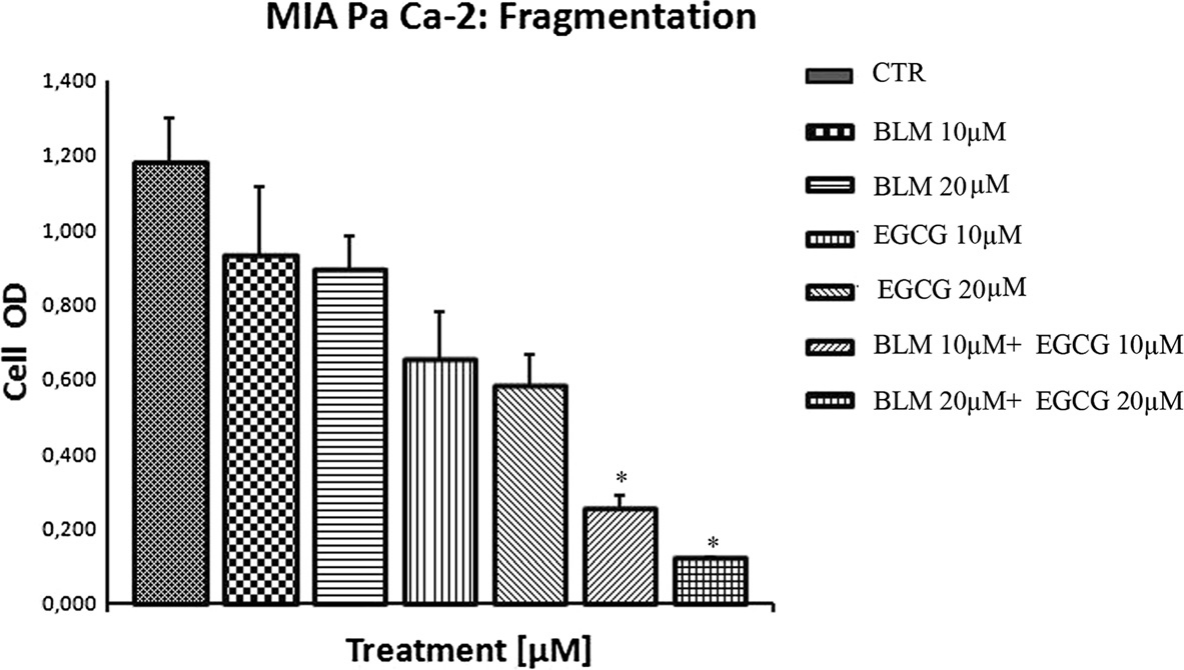
Effects of EGCG and BLM on MIA PaCa-2 cell death. Cell death detection using enzyme-linked immunosorbent assay at 72 h in MIA PaCa-2 cell lines in vitro when treated with EGCG alone, BLM alone, and both agents together. Data presented as mean ± standard deviation. ^∗^P < 0.001, by analysis of variance, compared with control

To investigate whether cytotoxic effects of BLM were due to necrosis or apoptosis, we performed an assay of released nucleosomes. Statistically significant increases in released nucleosomes when MIA PaCa-2 cells was treated with BLM (20 μM (110.6 %, 1.646 ± 0.01050) and EGCG (20 μM) (42.4 %, 1.113 ± 0.0015), and a statistically significant (*P* < 0.0001) decrease was observed when the cells were treated with the combination treatment (25 %, 0.586 ± 0.017) at 72 h (Fig. 3).

## Discussion

The present study investigated for the first time, the inhibitory effect of EGCG and BLM on human pancreatic cancer MiaPaca-2 cell growth. EGCG is the most widely studied catechin in green tea and has been found to inhibit the cell cycle and to induce apoptosis [17]. EGCG inhibits Hsp90 function by impairing Hsp90 association with chaperones in pancreatic cancer cell line Mia Paca-2 [36]. In addition, EGCG has anticancer effect in human pancreatic carcinoma cells by inhibition of both focal adhesion kinase and insulin-like growth factor-I receptor induction of CDK inhibitors, and inhibition of FAK and insulin-like growth factor-1 receptor [37]. Previous studies have proved the anticancer activity of EGCG and pterostilbene, including its activation of caspase-dependent apoptosis in pancreatic cancer [38]. Recent studies revealed that EGCG up-regulated JWA while decreased topoisomerase IIα expression in both human non-small cell lung cancer (NSCLC) cells and an NSCLC xenograft mice model [39]. It has been demonstrated that in human colorectal cancer cells, EGCG synergizes the therapeutic effect of cisplatin and oxaliplatin through autophagic pathway [40]. EGCG is considered an adjuvant for the treatment of ovarian cancer [41]. EGCG has antioxidant activities and resolved inflammation during BLM induced pulmonary fibrosis [42]. BLM is clinically used for cancer therapy [21]. BLM plays both central and coordinated roles in a network involving multiple pathways for signal transduction, chromosome remodeling, DNA/RNA binding and processing, DNA repair, cell cycle, and apoptosis [30, 32, 33]. The aim of our study was to evaluate the efficacy and synergism of EGCG and BLM in pancreatic cancer cells by different *in vitro* approaches. Our results indicate that EGCG and BLM have additive anti-proliferative effects in *vitro* by induction of apoptosis of MIA PaCa-2 cells. Additionally, we demonstrated that these compounds affect mitochondrial depolarization and cell cycle progression. This combination could represent a new strategy with potential advantages for treatment of pancreatic cancer. Although it is important to work to translate the effective *in vitro* dosages to effective *in vivo* models. To further investigate how these compounds affect the apoptosis and to elucidate the underlying mechanisms, further studies should be performed. Additional experiments by using different delivery systems such as microbubbles, nanospheres and cellular electroporation, will be needed to ameliorate their therapeutic effects and in particular to increase cellular permeabilization of BLM. In the future, we also plan to study the *in vivo* effects of EGCG and BLM in orthotopic pancreatic cancer mouse model.

## Conclusions

Our results indicate that EGCG and BLM have additive anti-proliferative effects in *vitro* by induction of apoptosis of MIA PaCa-2 cells. This combination could represent a new strategy with potential advantages for treatment of pancreatic cancer. To date, this is the first report published of the inhibitory effect of EGCG and BLM on human pancreatic cancer MIA Paca-2 cell growth.

## Materials and methods

### Cell culture

Human pancreatic cancer cell line MIA PaCa-2 stably transfected with red fluorescent protein (RPF) and Panc-1 were obtained from the American Type Culture Collection (ATCC) (Manassas, V, USA). Cells were cultured in RPMI 1640 (Gibco) supplemented with 10 % fetal bovine serum (FBS; Gibco, Long Island, NY, USA) and 1 % penicillin/streptomycin (Sigma Aldrich) at in humidified incubators at 37 °C under an atmosphere of 5 % C0_2_.

### Proliferation assay

The effect of EGCG and BLM on cell proliferation was determined by using TACS 3-(4,5-dimethylthiazol-2-yl)-2,5-diphenyltetrazolium bromide (MTT) cell proliferation assay (Trevigen, Githersburg). The cells (2000 per well) were incubated with EGCG alone, BLM alone or combination of EGCG and BLM, in triplicate in a 96-well plate and then incubated for 2, 4 and 6 days at 37 °C. A MTT solution was added to each well and incubated for 2 h at 37 °C. An extraction buffer (20 % SDS and 50 % dimethylformamide) was added, and the cells were incubated overnight at 37 °C. The absorbance of the cell suspension was measured at 570 nm using a microplate reader (DAS Technologies, Chantilly, VA). This experiment was repeated twice, and the statistical analysis was performed to obtain the final values.

### In Vitro apoptosis assay by flow cytometry

Cells were washed and suspended in 0.5 mL of PBS, and 1AL/mL YO-PRO-1, and propidium iodide was added. Cells were incubated for 30 min on ice and analyzed by flow cytometry (FACScan, Becton Dickinson, Franklin Lakes, NJ) by measurements of fluorescence emission at 530 and 575 nm. The apoptotic cells were stained with the green fluorescent dye YO-PRO-1 while necrotic cells were stained with propidium iodide. The apoptotic fraction was obtained by dividing the number of apoptotic cells by the total number of cells (minimumof 104 cells). Data were analyzed using CellQuest software (BectonDickinson). All data were reproduced at least third independent experiments.

### Mitochondrial depolarization

Cells were seeded at 3 × 10^5^ cells per well into 6-well plates and incubated overnight at 37 °C in the carbon dioxide incubator; 2 mM of JC-1 (Molecular Probes, Eugene, OR) was added to each well for 20 min at 37 °C. The cells were then washed with PBS and treated with a Medium as control or with EGCG (10 μM), EGCG (20 μM), BLM (10 μM), BLM (20 μM) or the combinations (10 μM EGCG plus 10 μM BLM), (20 μM EGCG plus 20 μM BLM) for 30 min. The cells were trypsinized, suspended in PBS, and run on the Coulter Elite Flow Cytometer. The excitation peak of JC-1 is 488 nm, and the approximate emission peak of the monometric and J-aggregate forms is 529 and 590 nm, respectively.

### Cell cycle analysis

The cells were seeded at 3 × 10^5^ cells per well into 6-well plates and incubated overnight at 37 °C in the carbon dioxide incubator. The cells were then washed with PBS and treated with a Medium as control or with EGCG (10 μM), EGCG (20 μM), BLM (10 μM), BLM (20 μM) or the combinations (10 μM EGCG plus 10 μM BLM), (20 μM EGCG plus 20 μM BLM) for and incubated for 24, 48, or 72 h. The cells were washed with phosphate-buffered saline (PBS), trypsinized, and incubated in ice-cold ethanol for 2 h. After incubation, the cells were washed with PBS and suspended in PBS plus 0.1 % Triton X-100 plus 100 μg/mL RNase A (Sigma Aldrich) plus 40 μg/mL propidium iodide (MP Biochemicals, Salon, OH) for 30 min in the dark. The cells were run on a Coulter Elite Flow Cytometer. Propidium iodide, when bound to nucleic acids, has an excitation maximum at 535 nm and an emission maximum at 617 nm. ModFit LT, version 3.0, software (Verity Software, Portland, ME) was used to analyze and categorize cell populations into cell cycle phases.

### DNA fragmentation assay

A Cell Death Detection ELISA^PLUS^ Kit (Roche Mannheim, Germany) was used to detect apoptosis in the cells. It is a sandwich enzyme immunoassay-based method used to detect the presence of nuclear DNA fragmentation. Mouse monoclonal antibodies directed against DNA and histones were used to recognize released nucleosomes after DNA nucleosomal fragmentation. The cells were seeded into 96-well plates at 10^4^ cells per well and allowed to incubate at 37 °C in the carbon dioxide incubator for 24 h for the cells to adhere. The cells were then washed with PBS and treated with a Medium as control or with EGCG (10 μM), EGCG (20 μM), BLM (10 μM), BLM (20 μM) or the combinations (10 μM EGCG plus 10 μM BLM), (20 μM EGCG plus 20 μM BLM) and allowed to incubate for 48 or 72 h. Lysis buffer was applied to the adherent cells, and the cells were centrifuged to produce a nucleosome-containing supernatant. The samples were transferred to a streptavidin-coated enzyme-linked immunoabsorbent assay microplate and incubated with anti-histone and anti-DNA antibodies. A peroxidase substrate was applied, causing a subsequent color change, proportional to the amount of nucleosomes captured in the antibody sandwich. The plates were read at 405 nm on a spectrophotometer.

### Statistical analysis

Data were analyzed using the Anova Test and expressed as mean values of at least three independent replications. Differences were considered to be highly statistically significant when p <0.01 and significant at p <0.05.

## Abbreviations

EGCG (−): −epigallocatechin-3-gallate
BLM: Bleomycin
